# Evaluation of cucumber seed germination vigor under salt stress environment based on improved YOLOv8

**DOI:** 10.3389/fpls.2024.1447346

**Published:** 2024-09-13

**Authors:** Zhengxin Fang, Qinglu Fan, Luxu Tian, Haoyu Jiang, Chen Wang, Xiuqing Fu, Xiaozhong Li, Meng Li, Shiyan Zhang, Yaben Zhang, Yingyue Li

**Affiliations:** ^1^ College of Engineering, Nanjing Agricultural University, Nanjing, China; ^2^ Cotton Research Institute, Xinjiang Academy of Agricultural and Reclamation Sciences, Shihezi, China; ^3^ College of Artificial Intelligence, Nanjing Agricultural University, Nanjing, China; ^4^ College of Mechanical Engineering, Yangzhou Polytechnic College, Yangzhou, China

**Keywords:** cucumber seed, phenotype acquisition system, target detection, YOLOv8 improvement, seed germination vigor assessment

## Abstract

Seed germination vigor is one of the important indexes reflecting the quality of seeds, and the level of its germination vigor directly affects the crop yield. The traditional manual determination of seed germination vigor is inefficient, subjective, prone to damage the seed structure, cumbersome and with large errors. We carried out a cucumber seed germination experiment under salt stress based on the seed germination phenotype acquisition platform. We obtained image data of cucumber seed germination under salt stress conditions. On the basis of the YOLOv8-n model, the original loss function CIoU_Loss was replaced by ECIOU_Loss, and the Coordinate Attention(CA) mechanism was added to the head network, which helped the model locate and identify the target. The small-target detection head was added, which enhanced the detection accuracy of the tiny target. The precision P, recall R, and mAP of detection of the model improved from the original values of 91.6%, 85.4%, and 91.8% to 96.9%, 97.3%, and 98.9%, respectively. Based on the improved YOLOv8-ECS model, cucumber seeds under different concentrations of salt stress were detected by target detection, cucumber seed germination rate, germination index and other parameters were calculated, the root length of cucumber seeds during germination was extracted and analyzed, and the change characteristics of root length during cucumber seed germination were obtained, and finally the germination activity of cucumber seeds under different concentrations of salt stress was evaluated. This work provides a simple and efficient method for the selection and breeding of salt-tolerant varieties of cucumber.

## 1 Introduction

At present, climate change, land degradation, and other environmental problems have brought great challenges to food security in China and the world. An in-depth analysis of crop genes to the phenotype of the environmental regulatory network, as well as the selection and breeding of novel high-yield, high-quality, green, and anti-stress varieties, can ensure the security of the national seed industry and food security. Cucumber (*Cucumis sativus* L.) is an annual climbing herbaceous plant of the Cucurbitaceae family, which is rich in nutritive value and adaptable; it is one of the widely grown vegetable crops in the world (Alsaeedi et al., 2019). Salt stress is one of the main factors leading to abiotic stress on plant growth and yield and one of the ecological crises facing agricultural production (Abdel-Farid et al., 2020). In China, the promotion of facility-based vegetable cultivation, especially greenhouse and greenhouse cultivation area, has expanded dramatically. However, given that soils in these facilities are seldom subject to rainfall, coupled with over-application of chemical fertilizers and long-term continuous cropping, soil secondary salinization (Li et al., 2014) has become a widespread problem, greatly affecting the efficiency of facility utilization and the sustainable development of facility vegetables. Salinity is becoming an increasingly prominent problem in modern agricultural production (Zhao et al., 2022). Salt stress can lead to excessive accumulation of salts in plants, causing ion toxicity, damaging the plant body, and leading to plant death (Zhou et al., 2021), which can seriously affect crop yield and quality.

Therefore, breeding salt-tolerant varieties that can grow and develop normally in saline environments has become a pressing issue in today’s agricultural production to improve the salt tolerance of crops. Seed germination vigor detection is crucial for the selection of superior varieties, and traditional seed germination vigor detection mostly relies on manual counting, manual measurement, and visual inspection, which feature low work efficiency, strong subjectivity, large error, and seed destruction; unfortunately, these methods are unable to satisfy the needs of modern breeding technology (ElMasry et al., 2019; Halcro et al., 2020; Hong et al., 2015; Nguyen et al., 2018). In recent years, image recognition has been continuously developed and widely used. The development and advantages of image recognition have been a significant focus in recent research. Yang et al. (2020) applied the ResNet50 algorithm to identify surface damage on wind turbine blades, showcasing the benefits of deep learning classification methods in image recognition. Mengbei et al. (2021) delved into image super-resolution reconstruction algorithms based on deep learning, demonstrating the satisfying effects of incorporating deep learning ideas into image processing. In recent years, the application of machine learning (ML) as a non-destructive testing technique has been widely used in the field of agricultural breeding (Rahman and Cho, 2016). Joshua Colmer et al. (2020) designed the SeedGerm system, which combines image acquisition and ML analysis modules to extract morphological features such as seed size, width, length, extent, and roundness to gain insight into the physiological process of seed germination, thereby enabling automated seed imaging and high-throughput germination analysis. Medeiros et al. (2020) utilized Fourier transform near-infrared spectroscopy and X-ray imaging to merge data for nondestructive seed detection and accurate quality classification. Škrubej et al. (2015) used six ML classification algorithms such as Support Vector Machines (SVMs) and Artificial Neural Networks (ANNs) to identify germinating tomato seeds and accurately calculate the germination rate. Lurstwut and Pornpanomchai (2017) developed a rice seed germination evaluation system (RSGES) based on ANN to achieve an accurate assessment of the germination status of rice in Thailand. Traditional ML is suitable for small-scale dataset processing and specific shallow feature information extraction tasks. However, it has difficulty in adapting to feature recognition in different environments and meeting the extraction of complex phenotypic gesture feature recognition in the seed germination process.

In recent years, deep learning techniques have become an effective method for feature detection; they have made significant breakthroughs in the field of target detection and have been applied to agricultural detection in a large number of applications (Yang et al., 2021). The YOLO series model, as a convolutional neural network, is widely used in target detection and localization due to its advantages such as real-time detection and high accuracy. Touko Mbouembe et al. (2024) realized the detection of tomato fruits in the natural environment using an improved SBCS-YOLOv5s model based on YOLOv5; Li et al. (2024) proposed a weed detection model, YOLOv7-FWeed, which provides an accurate and efficient solution for weed detection in soybean fields; Zhang et al. (2023) proposed a new model, MDY7-3PTB, that combines the fast detection capabilities of DeepLabv3+ and YOLOv7; it realizes the process of segmenting the tea buds, detecting them, and localizing them to accurately identify the tea buds’ picking points. Although the YOLO algorithm is widely used in agriculture, there are few studies on seed germination vigor detection, and the deep learning technique greatly reduces the modeling process of seed discrimination by eliminating the need for feature extraction and morphological processing of the image during detection (Bai et al., 2023). Fu et al. (2022) evaluated the germination vigor and salt tolerance of wheat seeds using the YOLOv4 model; Jiang et al. (2023) developed the YOLOv8-Peas model to realize the determination of drought resistance of different varieties of pea seeds. Rapid identification and localization of seed germination by deep learning can provide a new solution for seed germination vigor detection. In this study, using the improved YOLOv8 algorithm YOLOv8-ECS based on the seed germination phenotype collection system, we carried out a cucumber germination test in a salt-stressed environment and successfully extracted the phenotypic characteristics of cucumber seed germination. Moreover, we analyzed the characteristics of the changes in the germination parameters, such as the germination rate, germination index, and root length, and evaluated the vigor of the cucumber seed germination under the environment of different concentrations of salt stress. According to the pre-test, cucumber seeds begin to sprout their embryonic roots around 24 hours of incubation, and young leaves sprout after about 48 hours, in order to reduce the recognition difficulty of the algorithm, we are currently only investigating the cucumber seed sprouting phenological feature extraction within the first 48-hour time period. The system consisted of three main areas:

1. The seed germination phenotype acquisition system was equipped with the growth conditions required for seed germination, and could realize the complete cycle image information acquisition and automatic storage of the whole process of seed germination.
2. The cucumber seed germination test under salt stress environment was carried out in the collection system, and NaCl aqueous solution was used to provide the salt stress environment. Five concentrations of NaCl solution (i.e., 30, 60, 90, 120, and 150 mmol/L) were selected for the test, and deionized water was used as the control (CK). Images of the germination of cucumber seeds under different concentrations of NaCl solution were acquired, and the cucumber seed germination dataset was constructed.
3. Based on the YOLOv8-n model, the original model loss function CIOU_Loss was replaced by the ECIoU_Loss loss function The CA mechanism was added to the model head, and a small target detection head was added to obtain the YOLOv8-ECS model for the detection of cucumber seed germination, which greatly enhanced the accuracy of phenotypic micro-target feature recognition and extraction in the process of cucumber seed germination. The improved model was used to detect the germination status of cucumber seeds under different concentrations of salt stress environments, and further calculated and analyzed germination vigor indexes such as germination rate, germination index and root length.

## 2 Materials and methods

### 2.1 Phenotype acquisition system

**Figure 1:**
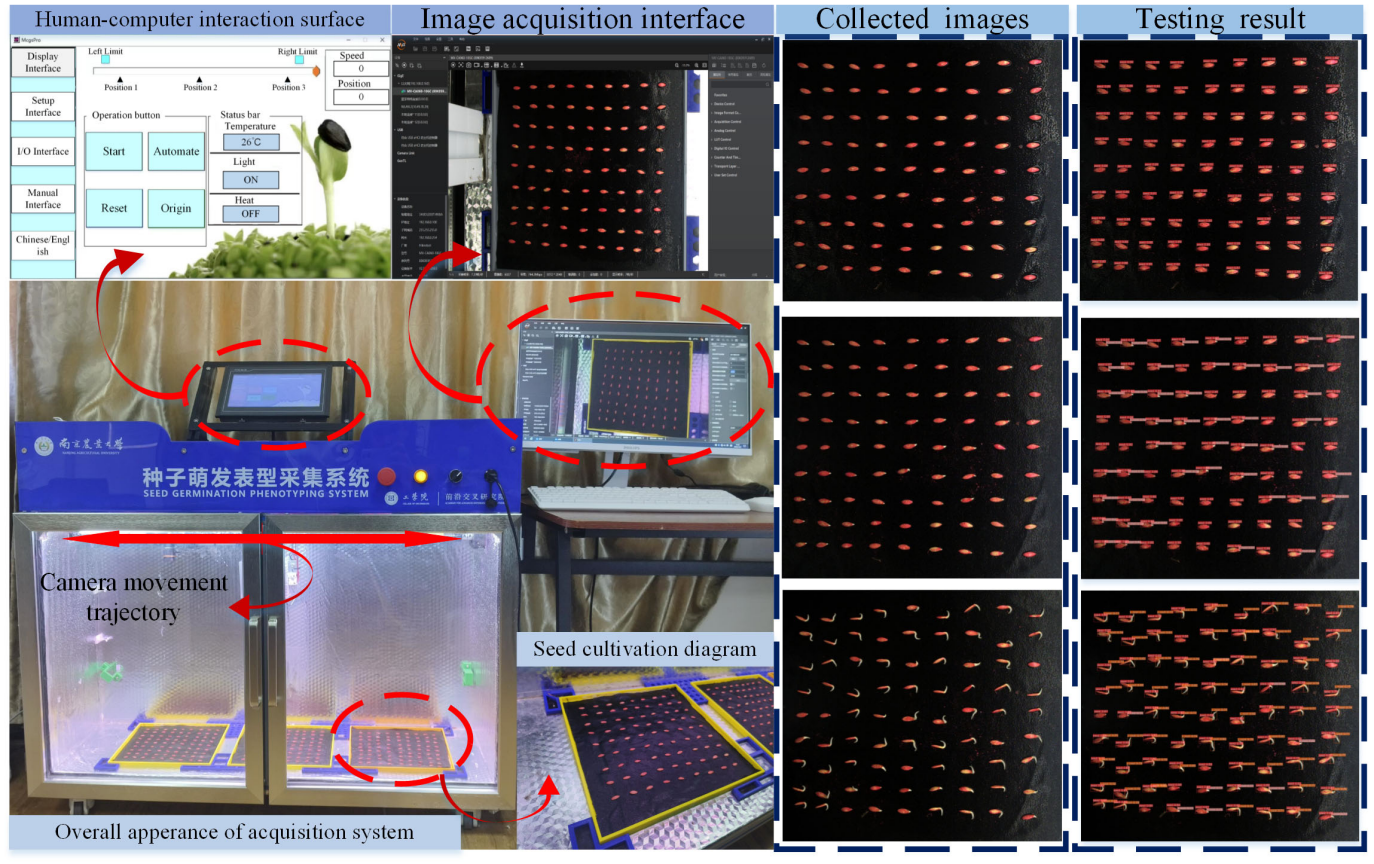
Seed germination phenotype acquisition system.

The seed germination phenotype acquisition system is shown in **Figure 1**, which consisted of three parts: seed germination culture bin, image acquisition system, and human–computer interaction module. The incubator was equipped with temperature adjustment and light adjustment functions, which could adjust the temperature (15 °C-50 °C) and light in the incubator in real time according to the environmental status, thereby maintaining a relatively stable environment for seed germination. Three incubation trays were placed in the incubator, which could be used to carry out three concentration stress tests simultaneously. The transmission mechanism equipped with Hikvision RGB camera (MV-CS060-10GC) could move back and forth along the linear guideway to dynamically collect sprouting images at a fixed point. Software control enabled the timed collection of sprouting images. The collected images were saved to the corresponding folder through PLC program control, and the collected sprouting images were preprocessed and the dataset was produced. A suitable target detection algorithm was selected to train the produced dataset, and the optimal model was obtained by comparing the detection accuracy and other indexes of each model. Thus, an accurate and rapid detection result of seed sprouting vitality was obtained, and an automatic analysis of the seed sprouting phenotypic data could be realized.

### 2.2 Data collection and dataset construction

**Figure 2:**
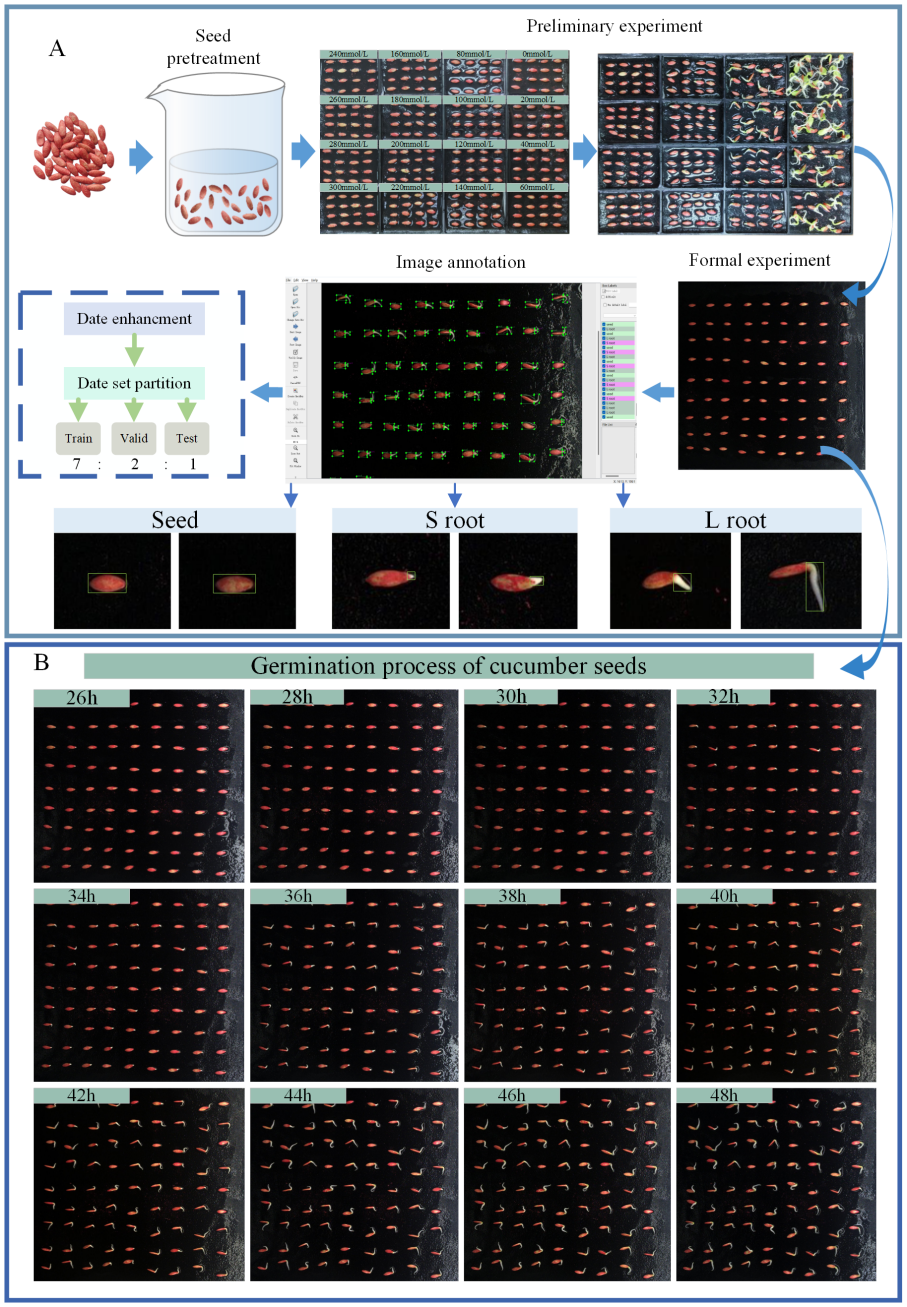
Experimental procedures and dataset construction. **(A)** Experimental pre-treatment and dataset construction. **(B)** Image of cucumber seed germination process.

The experiment was conducted at Nanjing Agricultural University, and the test variety was ZhiLv 0135 cucumber seed purchased from Nanjing Green Collar Seed Industry Co. Full, intact, and uniformly sized cucumber seeds were selected for salt stress pre-tests in 16 small compartments of culture trays before the formal test, in which three layers of black filter paper were spread in the culture trays. The concentrations of 0, 20, 40, 60, 80, 100, 120, 140, 160, 180, 200, 220, 240, 260, 280, and 300 mmol/L NaCl were applied. To create an aqueous solution, we soaked the seeds in deionized water for 6 h. After absorbing the water on the surface of the seeds, the cucumber seeds were spotted in a culture plate, and 16 (4×4) seeds were placed in each small cell and placed into the germination system for seed germination cultivation. The temperature of the system was maintained at 26°C (± 1°C). The mass of the culture trays was weighed at regular intervals during the germination process using the weighing method, and the corresponding solution was replenished according to the change in mass in order to ensure that the concentration of NaCl in the stress solution was relatively stable. The pre-tests were carried out for 72 h. The NaCl concentration in the 160 mmol/L solution was lower than that in the 160 mmol/L solution in the pre-tests. Moreover, seed germination was not observed in the 160 mmol/L concentration and above. Finally, we chose five NaCl concentrations of 30, 60, 90, 120, and 150 mmol/L for the formal test. The formal test seeds were treated in the same way as the pre-test seeds. A total of 81 (9×9) seeds were placed in each culture plate, and deionized water was used as the control (CK). Each concentration was repeated three times. The test parameters are shown in **Table 1**, and the specific test steps are shown in **Figure 2A**.

**Table 1:** Parameters of emergence test.

Parameters	Numerical value
Number of test seeds	1458
Temperature	26°C ( ± 1°C)
Concentration of NaCl (mmol/L)	0 (CK), 30, 60, 90, 120, 150
Number of replicates per concentration	3
Time of germination	48h
Image acquisition interval	30min
Number of acquired images	1728

**Figure 3:**
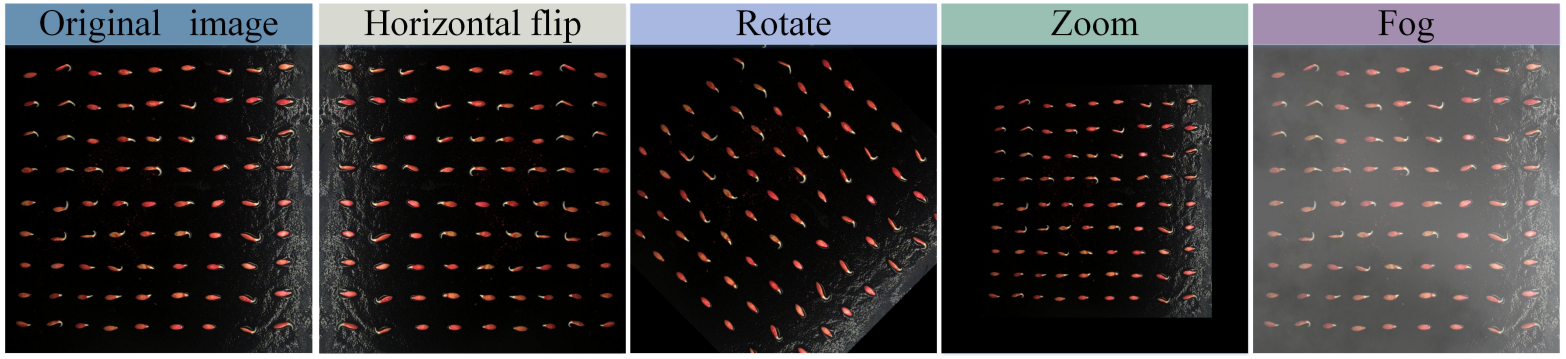
Data set enhancement.

During the germination process, the camera was set to collect images every 30 min, and some of the germination images collected are shown in **Figure 2B**, which were observed as the phenotypic gesture changed during the germination of cucumber seeds. A total of 1,728 germination images were collected by applying the phenotypic collection system. Since the cucumber seed sprouting phenomenon was not obvious before 24 hours, we eliminated the images where the sprouting phenomenon was not obvious in the first 24 hours and selected 500 images among the remaining images to construct the dataset for the model. We took the root length elongation to half of the seed length as the germination standard and used LabelImg software to label the information in the images into three types: seed, S root (root length not reaching half of the seed length), and L root (root length more than half of the seed length), and some of the labeling examples are shown in **Figure 2A**. The file marked with LabelImg is stored in xml format, which cannot be used for training directly. In order to meet the training requirements of the model, we use the relevant python script to convert the xml file into a txt file, which contains the normalized width, height, and center coordinates of the bounding box, as well as the category labels. After labeling, to enhance the recognition ability and adaptability to complex environments, prevent overfitting of the model, and improve the robustness of the model, we performed image enhancement operations such as horizontal flipping, rotating, scaling, and adding noise (**Figure 3**). We processed 100 images with each enhancement to get 500 processed images. Finally, 1000 images were obtained, which were classified into the training set, validation set, and test set according to the ratio of 7:2:1. After dividing the dataset, the training set, validation set and test set have 700, 200 and 100 images respectively.

#### 2.2.1 Design of the YOLOv8-ECS algorithm

The You Only Look Once (YOLO) family is a well-known single-stage target detection network. Since Redmon et al. (2016) first proposed YOLOv1, the YOLO family has established an important position in the field of target detection. Released in 2023, YOLOv8 represents the current state-of-the-art in target detection, image classification, and instance segmentation. As the latest version of the YOLO series, it offers five different scale models, namely, YOLOv8-n, YOLOv8-s, YOLOv8-m, YOLOv8-l, and YOLOv8-x, to accommodate diversified inspection needs. In constructing its backbone network and Neck part, it draws on the design concept of YOLOv7 ELAN, which enhances the gradient flow by adopting the C2f structure instead of the C3 structure, while adjusting the number of channels according to the different scales of the model, which significantly improves the performance. In the Head section, YOLOv8 introduces a novel decoupled head structure, which is a significant change compared with YOLOv5. It treats classification and regression tasks separately using two different loss functions: the classification task uses binary cross entropy (BCE loss), whereas the regression task combines distribution focus loss (DFL loss) and complete intersection union loss (CIoU loss).

**Figure 4:**
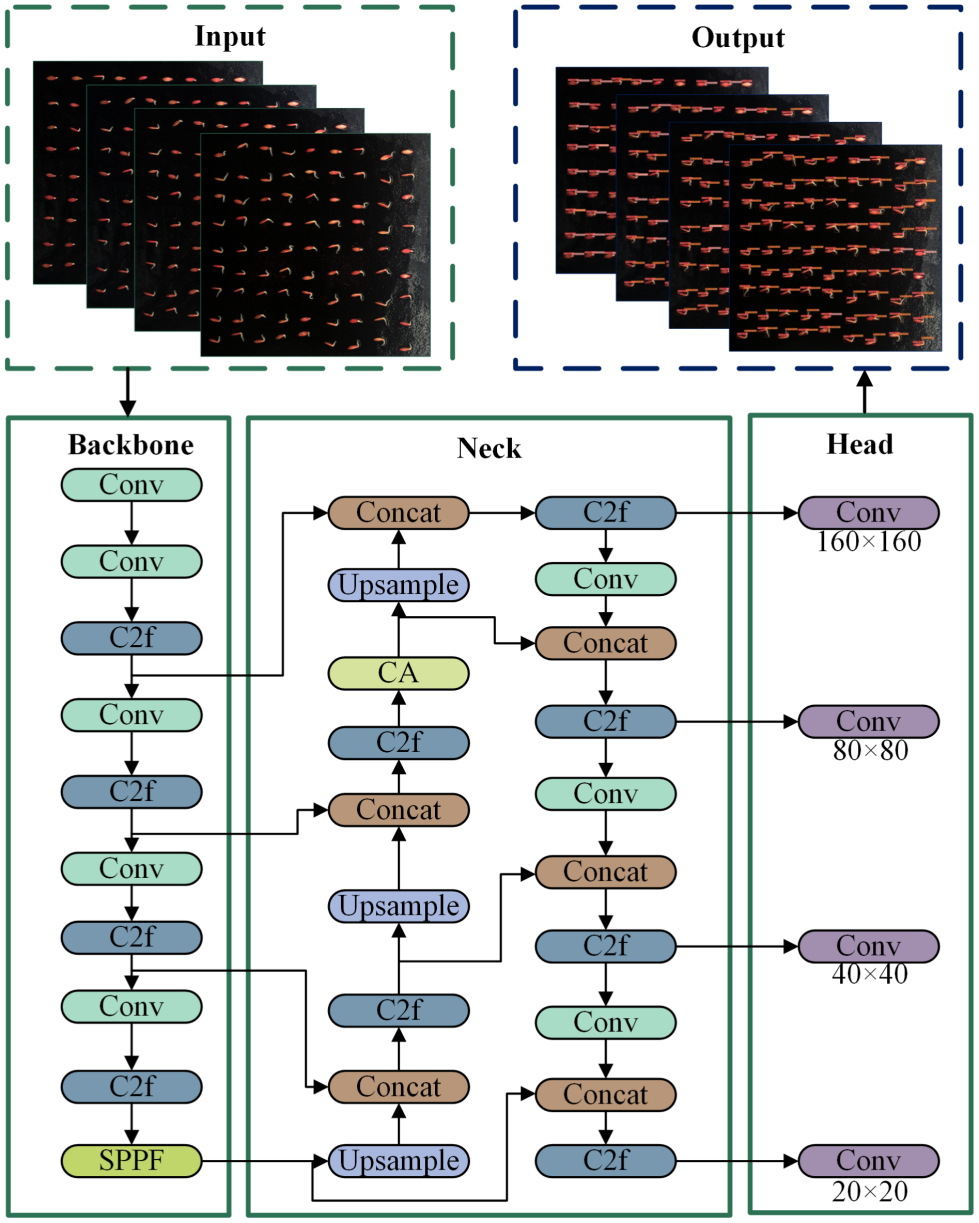
Structure of the YOLOv8-ECS model.

Although the YOLOv8 network demonstrates excellent performance in terms of accuracy and speed of target detection, it still faces some challenges when detecting tiny targets in complex environments. To enhance the ability of the model in identifying and extracting tiny phenotypic features during cucumber seed germination, we chose YOLOv8-n as the base model. We customized and improved it to obtain a model specifically designed for the detection and identification of tiny targets during the germination stage of cucumber seeds; this model was named YOLOv8-ECS. The structure of the model is shown in **Figure 4**. The YOLOv8-ECS model mainly consisted of five components: Input, Backbone, Neck, Head, and Output. First, we replace the original CIoU loss function with the ECIoU loss function, which facilitated the adjustment of the prediction frames and accelerated the regression rate. Second, we added a CA mechanism in the Neck part, which strengthened the localization and recognition capability of the model for specific targets. Lastly, for the recognition of tiny phenotypic features in the germination of cucumber seeds, we added a small-target detecting head, which effectively improved the model’s detection accuracy.

#### 2.2.2 EfficiCIoU-Loss loss function

In the field of target detection, the detection process usually consists of two key components: localization and recognition. The accuracy of localization mostly relies on the modulation of the loss function, and several novel loss functions have been proposed (Zheng et al., 2020; Vakili et al., 2023; Sun et al., 2020). Intersection Over Union (IoU) is widely used in bounding box regression as a mainstream metric to measure the similarity between predicted and real bounding boxes. The IoU loss function was developed to improve the effectiveness of IoU; however, the IoU loss function may fail when the prediction frame does not overlap with the true frame. To address this challenge, researchers have developed various IoU-based evaluation mechanisms to improve the limitations of the original IoU loss function and significantly enhance its robustness. Among these methods, including loss functions such as Generalized IoU (GIoU) (Rezatofighi et al., 2019), Distance Intersection over Union (DIoU), and Complete Intersection over Union (CIoU) (Zheng et al., 2020), all of them have achieved remarkable results in the field of target detection. However, there is still room for optimization. In particular, CIoU_Loss is one of the best-performing boundary regression loss functions, which integrates three key geometric factors, namely, overlap area, centroid distance, and aspect ratio. It combines the IoU, the Euclidean distance, the corresponding aspect ratio, and the angle to evaluate the degree of overlap between the target and the real frame, which is calculated by the following formulas:


(1) $$R_{\text{CIoU}}=\frac{\rho^{2}(b,b^{ɡt})}{c^{2}}+\alpha v$$

(2) $$v=\frac{4}{\pi^{2}}{\left(\text{arctan}\frac{\omega^{\text{ɡt}}}{h^{\text{ɡt}}}-\text{arctan}\frac{\omega }{h}\right)}^{2}$$

(3) $$L_{\text{CIoU}}=1-\text{IoU}+\frac{\rho^{2}b,b^{ɡt}}{c^{2}}+\alpha v$$

The width and height of the prediction box during regression cannot be increased or decreased at the same time. In cases where the width–height differences and their confidence levels are not real, once the model converges to a line-to-line ratio between the width and height of the predicted and real frames, it sometimes prevents the model from optimizing the similarity effectively. To solve the problem of CIOU_Loss, we calculate the loss function EIOU_Loss by splitting the aspect ratio influence factor on the basis of CIOU_Loss to ensure that the aspect of the predicted and real frames can be optimized effectively. When distant edges are present, the computation of EIOU_Loss may slow down but will not converge prematurely. To cope with this problem, we propose a new augmented loss function, ECIOU_Loss, which facilitates the adjustment of prediction frames and accelerates the regression rate. ECIOU_Loss is based on two loss functions: CIOU_Loss and EIOU_Loss. First, the predicted aspect ratio is adjusted by CIOU_Loss until it converges to a suitable range. Each edge is then carefully tuned by EIOU_Loss until it converges to the correct value. ECIOU_Loss is calculated as follows.


(4) $$ECIOU_{\text{Loss}}=1-\mathrm{IOU}+\text{αv}+\frac{\rho^{2}\left(b^{ɡt},b\right)}{c^{2}}\;+\;\frac{\rho^{2}\left(h^{ɡt},h\right)}{c_{h}^{2}}+\frac{\rho^{2}\left(\omega^{ɡt},\omega \right)}{c_{\omega }^{2}}$$

#### 2.2.3 CA mechanism

**Figure 5:**
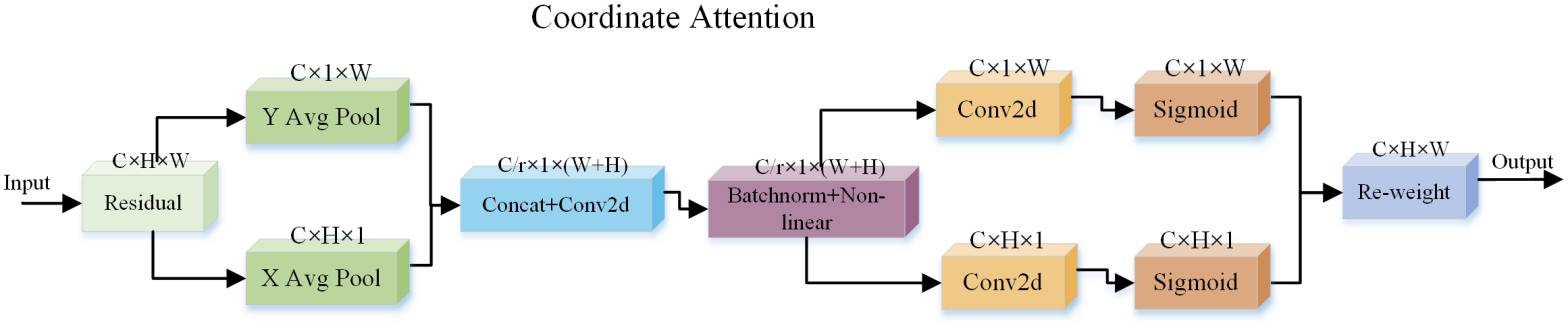
Coordinate attention mechanism.

Attention Mechanism (AM) (Niu et al., 2021; Guo et al., 2022) is an approach inspired by studies of human vision. In the field of cognitive science, as a result of the limitations of information processing, humans selectively focus on a portion of information while ignoring others. To rationally utilize the limited visual information processing resources, humans need to select specific regions and focus their attention on these regions (Sun et al., 2021). AM consists of two main aspects: selecting the parts that require attention and allocating limited information processing resources to the important information. Currently popular in deep learning networks is the SE (Hu et al., 2018) attention mechanism, which achieves a significant performance improvement by computing channel attention on top of 2D pooling, and the computational cost is relatively low. However, SE attention only considers the coding of information between channels and ignores the importance of location information. To solve this problem, Hou et al. (2021) proposed the Coordinate Attention (CA) mechanism, which is able to encode horizontal and vertical location information in the channel attention, thereby enabling the network model to focus on a wide range of location information without adding excessive computational cost. The network structure of the CA mechanism is shown in **Figure 5**.

#### 2.2.4 Small-target detection head

The original YOLOv8 has three detection heads: P3/8 corresponds to a detection feature map size of 80×80, detecting targets with a size of 8×8 or more; P4/16 corresponds to a detection feature map size of 40×40, detecting targets with a size of 16×16 or more; and P5/32 corresponds to a detection feature map size of 20×20, detecting targets with a size of 32×32 or more. Problems such as missed detection or poor results can occur in the detection of tiny targets for seed germination. As shown in **Figure 4**, we added a P2/4 detection head to the original model, which corresponded to a 160×160 detection feature map, and it could be used to detect more than 4×4 targets to enhance the detection accuracy of tiny targets. This improvement slightly increased the amount of model computation. However, by obtaining additional feature information from tiny targets, it greatly improved the detection ability of small targets and effectively reduced the misdetection and omission of objects at different scales.

### 2.3 Model training parameter setting

In this study, the environment configuration used was as follows: we chose an Intel(R) Xeon(R) Gold 6248R @ 3.00GHz processor equipped with an NVIDIA GeForce RTX3090 graphics card. The deep learning modeling framework used was Pytorch 2.0.0 and Python 3.8, the CUDA version chosen was 11.7, and the operating system was Windows 11. To ensure fairness and comparability of model effects in the study, we did not use pre-trained weights for model training in all ablation and comparison trials. In the training phase, we resized the input image to 640 × 640 pixels and set the total number of iterations to 300. **Table 2** provides details about the important parameter settings during the training process.

**Table 2:** Model training parameters.

Parameters name	Parameters value
Epoch	300
Batch size	8
Image size	640×640
NMS IoU	0.7
Learning Rate	0.01
Momentum	0.937
Weight Decay	5×10^-4^

### 2.4 Model evaluation indexes and cucumber seed germination vigor evaluation indexes

We chose Precision, Recall, Average Precision (AP), and mean Average Precision (mAP) as the evaluation indexes of model detection accuracy, and their formulas were as follows:


(6) $$\text{Precision}=\frac{\text{TP}}{\text{TP}+\text{FP}}$$

(7) $$\text{Recall}=\frac{\text{TP}}{\text{TP}+\text{FN}}$$

(8) $$\text{AP}=\int_{0}^{1}\;P\left(R\right)dR$$

(9) $$\text{mAP}=\frac{\sum_{n=1}^{N}AP_{n}}{N}$$

where True Positive (TP) denotes the number of correctly identified cucumber seed and radicle samples, False Positive (FP) denotes the count of incorrectly identified positive cucumber seed and radicle samples, and False Negative (FN) denotes the number of missed cucumber seed and radicle samples.

We chose seed root length elongation to half of the seed length as a marker of cucumber seed germination. We utilized germination rate, germination index, and root length as indicators of germination vigor.


(10) $$\text{Germination rate}\left(\%\right)=\frac{N_{s}}{N}\times 100\%$$

(11) $$\text{Germination index}\left(\%\right)=\sum (G_{t}\;/D_{t})$$

(12) $$\begin{array}{c} \text{Mean}\;\text{root}\;\text{lenɡth}\\ \;=\text{total}\;\text{root}\;\text{lenɡth}/\text{number}\;\text{of}\;\text{ɡerminated}\;\text{seeds} \end{array}$$

where *N_S_* denotes the number of germinated seeds in s hours, *N* denotes the total number of seeds, *G_t_* is the number of germination in t hours, and *D_t_* is the time of germination.

## 3 Results

### 3.1 Ablation test

**Figure 6:**
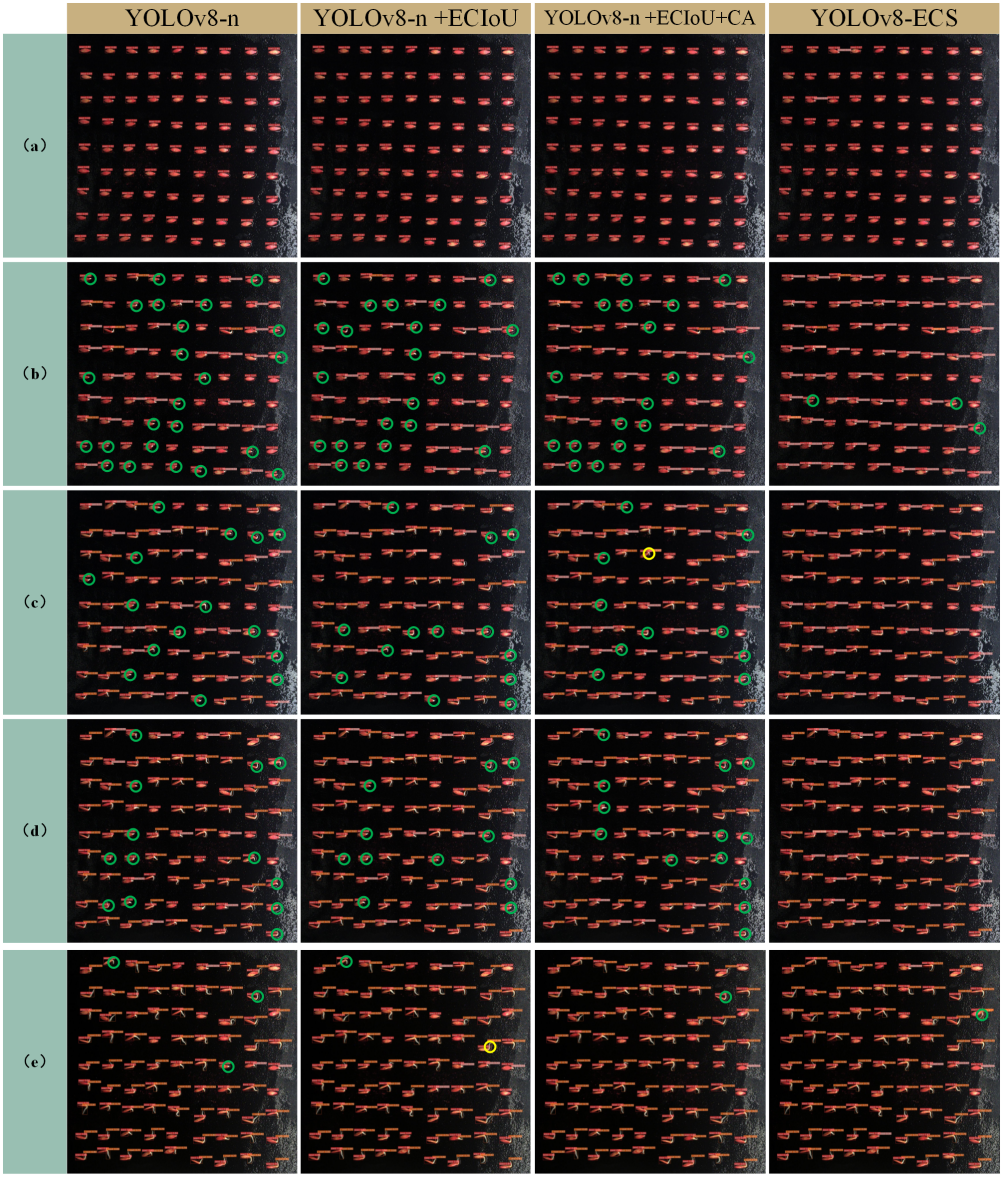
Comparison of ablation test effects. **(A–E)** Denote the results of the experiments for different germination periods.

To verify the effectiveness of the improved model, we conducted an ablation test, using the model detection accuracy as a measure of the index price, and the test results are shown in **Table 3**. First, the original loss function CIoU_Loss was replaced by ECIoU_Loss, and the model mAP improved by 0.5%. The CA mechanism was added to the head, and mAP was further improved to 93.4%. Finally, the small-target detection head was added to obtain the YOLOv8-ECS model, and mAP reached 98.9%. To further highlight the visualization advantages of the improved model, we chose five images of different sprouting periods (**Figures 6A–E**) for detection analysis, where the part circled in green indicates leakage detection, and the part circled in yellow indicates repetitive detection. No leakage was detected in the seeds. However, for the detection of the roots, in the beginning of the sprouting period (b), YOLOv8-n had a high number of missed detections, and the number of missed detections decreased slightly after adding the ECIoU loss function and the CA mechanism. The addition of the small-target detection head strengthened the ability to capture tiny targets, with no missed detections. The number of missed detections decreased in each model at mid-emergence (c) and (d), but missed detections still occurred in the first three models. No missed detections occurred in YOLOv8-ECS. In the later stages of germination (e), the models essentially stopped missing detections. The ablation test clearly showed that our improved YOLOv8 model had high accuracy for cucumber seed germination phenotypic feature recognition, which verified that our changes were effective.

**Table 3:** Comparison of ablation test parameters.

Model	Precision (%)	Recall (%)	mAP50 (%)
YOLOv8-n	91.6	85.4	91.8
YOLOv8-n+ECIoU	92.1	85.5	92.3
YOLOv8-n+ECIoU+CA	92.2	88.2	93.4
YOLOv8-ECS	97.2	97.4	98.9

### 3.2 Comparative tests

Using the same training environment and experimental parameters, we compared the detection results of different network models. **Table 4** shows the performance differences exhibited by the detection models using various modules, which mainly examined the mAP values of the models. The mAP50 value of detection using YOLOv8-n as the training model was higher than that of the other base models, so we chose YOLOv8-n as the base model and improved it to obtain the YOLOv8-ECS model.

**Table 4:** Comparison of detection performance of different models.

Model	Precision (%)	Recall (%)	mAP50 (%)
Faster R-CNN	89.5	86.3	89.3
YOLOv3	85.1	78.9	86.7
YOLOv4	88.7	89.1	89.6
YOLOv5s	93.4	92.1	90.9
YOLOv7-tiny	94.2	88.3	90.1
YOLOv8-n	91.6	85.4	91.8
YOLOv8-ECS	97.2	97.4	98.9

**Figure 7:**
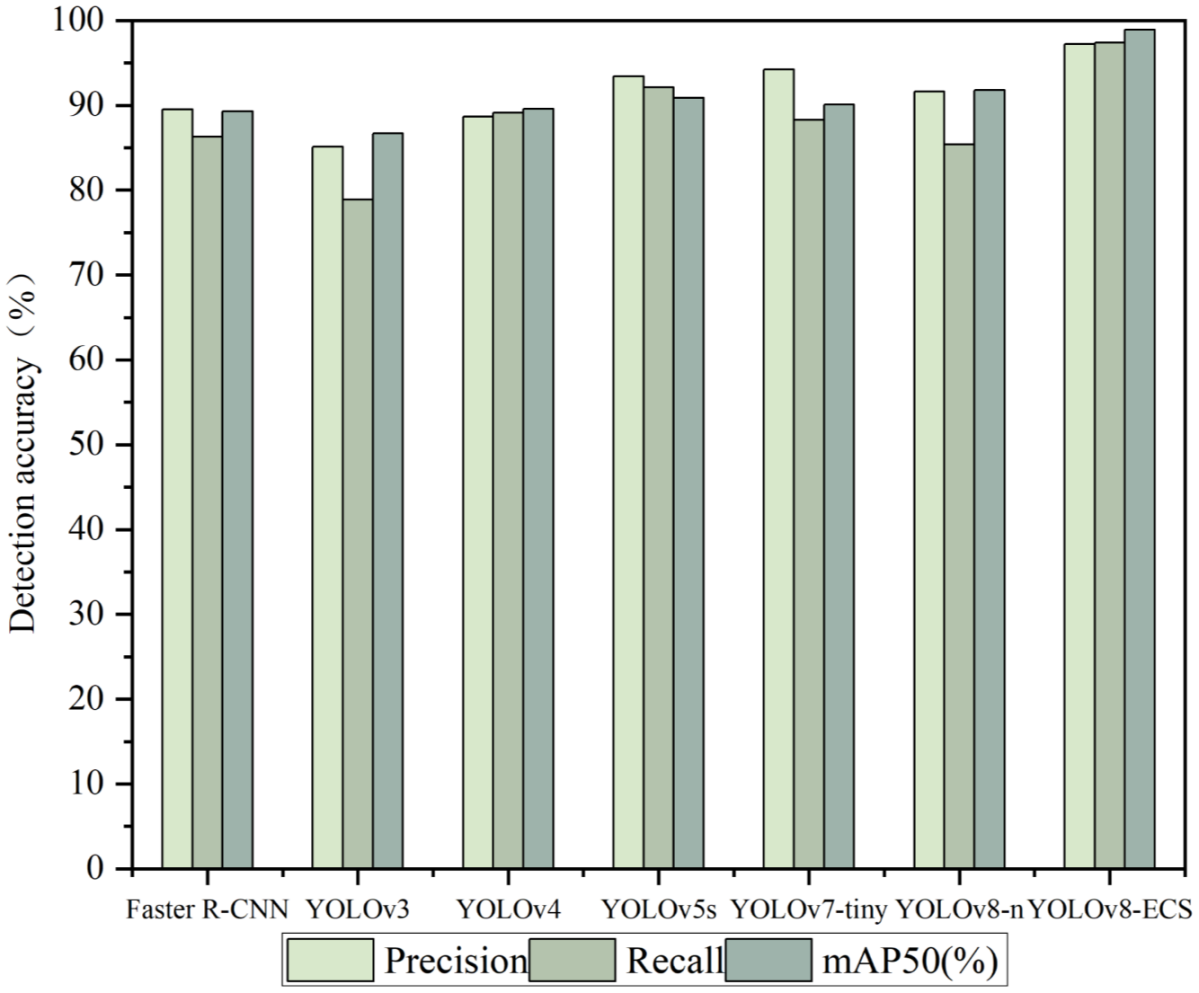
Histogram of comparison of different models.

To show the performance of the models in these evaluation metrics, we plotted the histograms of the detection accuracy of the different models, and the results are shown in **Figure 7**. Among the tested models, YOLOv8-ECS had the highest Precision, Recall, and mAP50 (%). Thus, the YOLOv8-ECS model demonstrated good accuracy and applicability in cucumber seed germination state detection.

### 3.3 Evaluation of seed germination vigor of cucumber under different concentrations of salt stress environment

**Figure 8:**
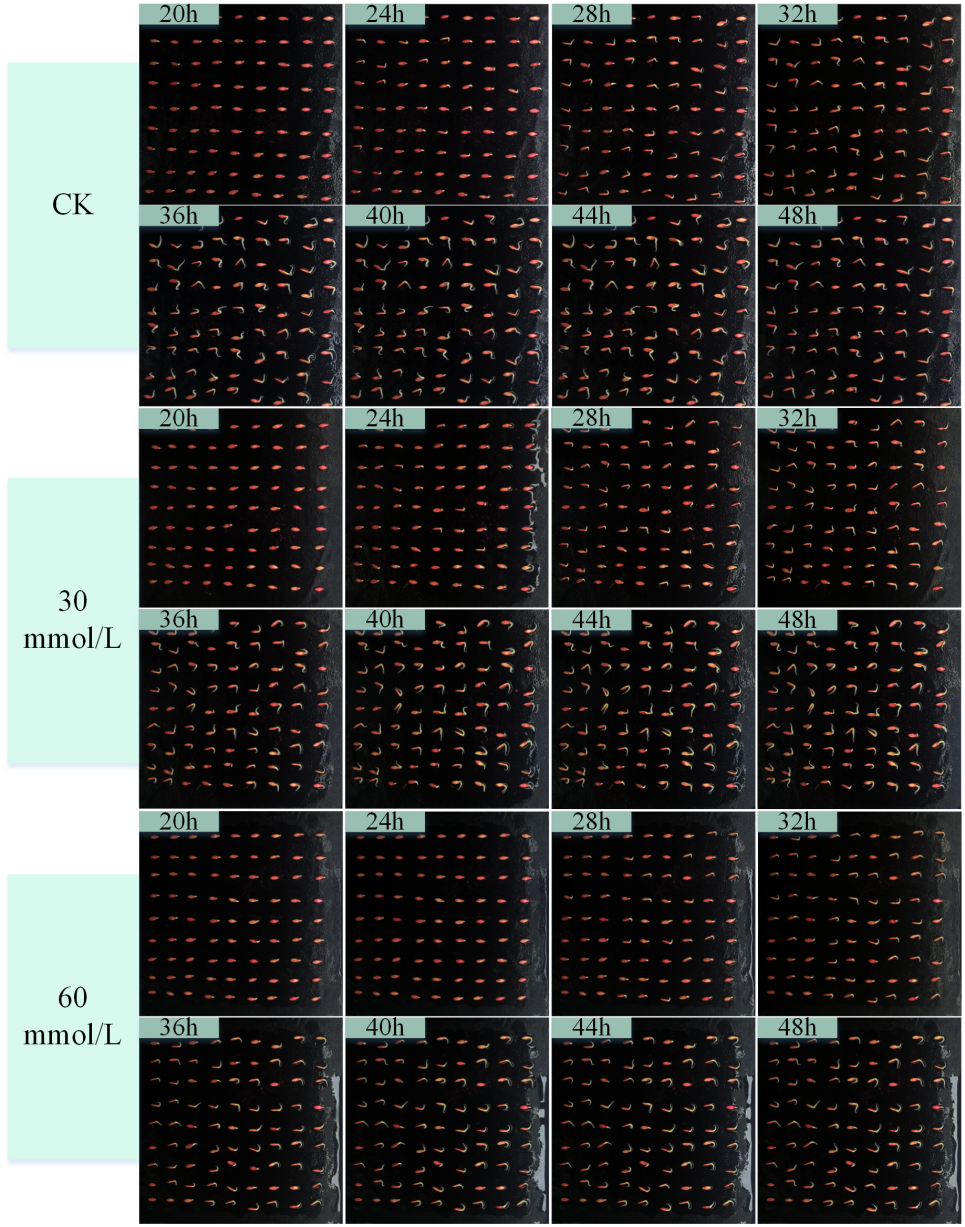
Images of the germination process of cucumber seeds in deionized water and different concentrations of NaCl solution (0, 30, 60mmol/L).

**Figure 9:**
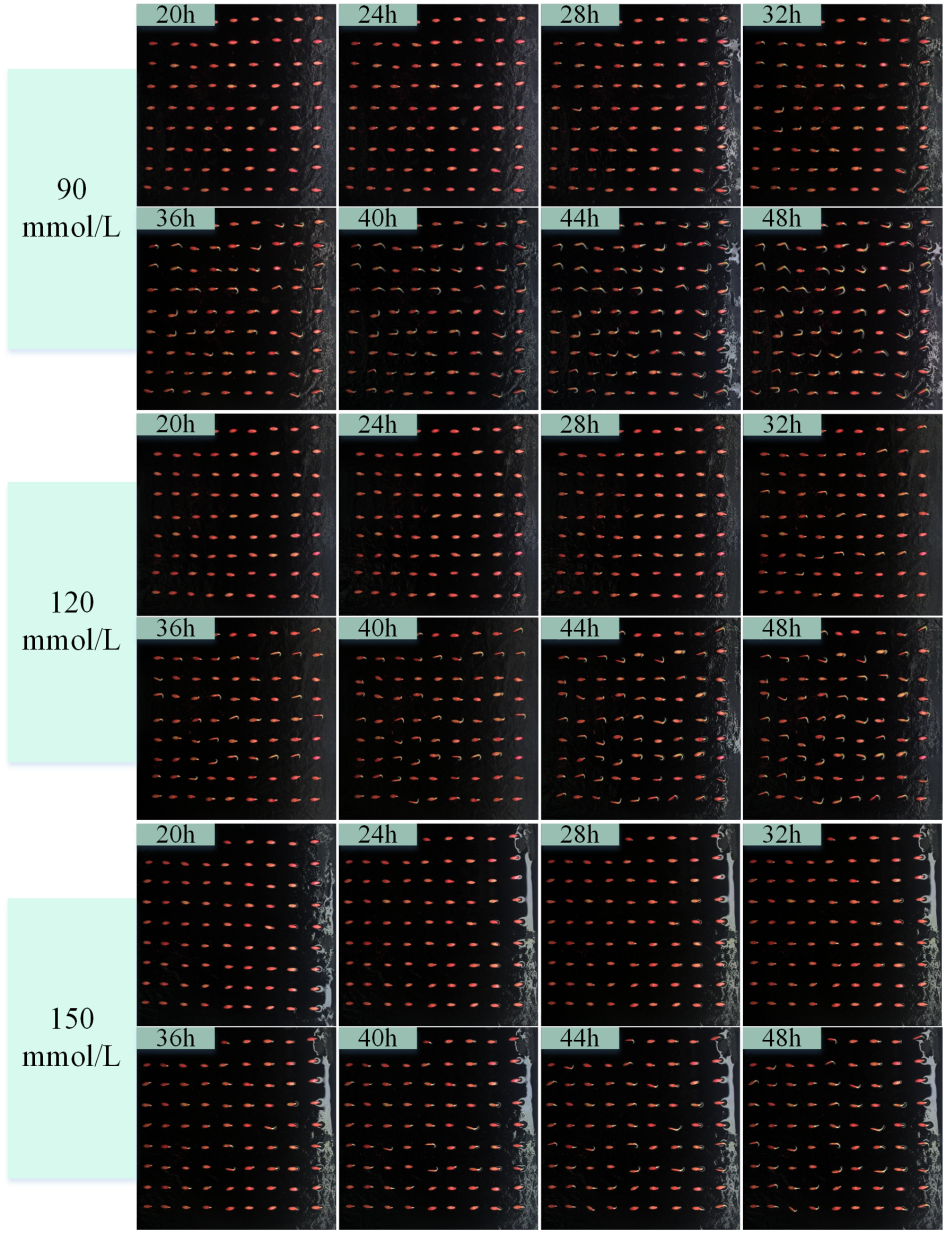
Images of the germination process of cucumber seeds in different concentrations of NaCl solution (90, 120, 150mmol/L).

Salt stress is one of the main factors leading to abiotic stress on plant growth and yield and one of the ecological crises facing agricultural production (Song et al., 2023). We carried out a cucumber seed germination test under salt stress environment, according to the pre-test germination environment. We set five concentrations of NaCl solution at 30, 60, 90, 120, and 150 mmol/L, and deionized water was used as a control (CK). We obtained a continuous image of the complete cycle of cucumber seed germination in different salt solutions. **Figure 8** shows the characteristics of cucumber seed germination in different concentrations of NaCl solution over time. The acquired images of the germination cycle were used for target detection with the YOLOv8-ECS model. The germination rate and germination index patterns and trends of cucumber seeds in different concentrations of NaCl solution were calculated, as shown in **Figures 8** and **9**.

**Figure 10:**
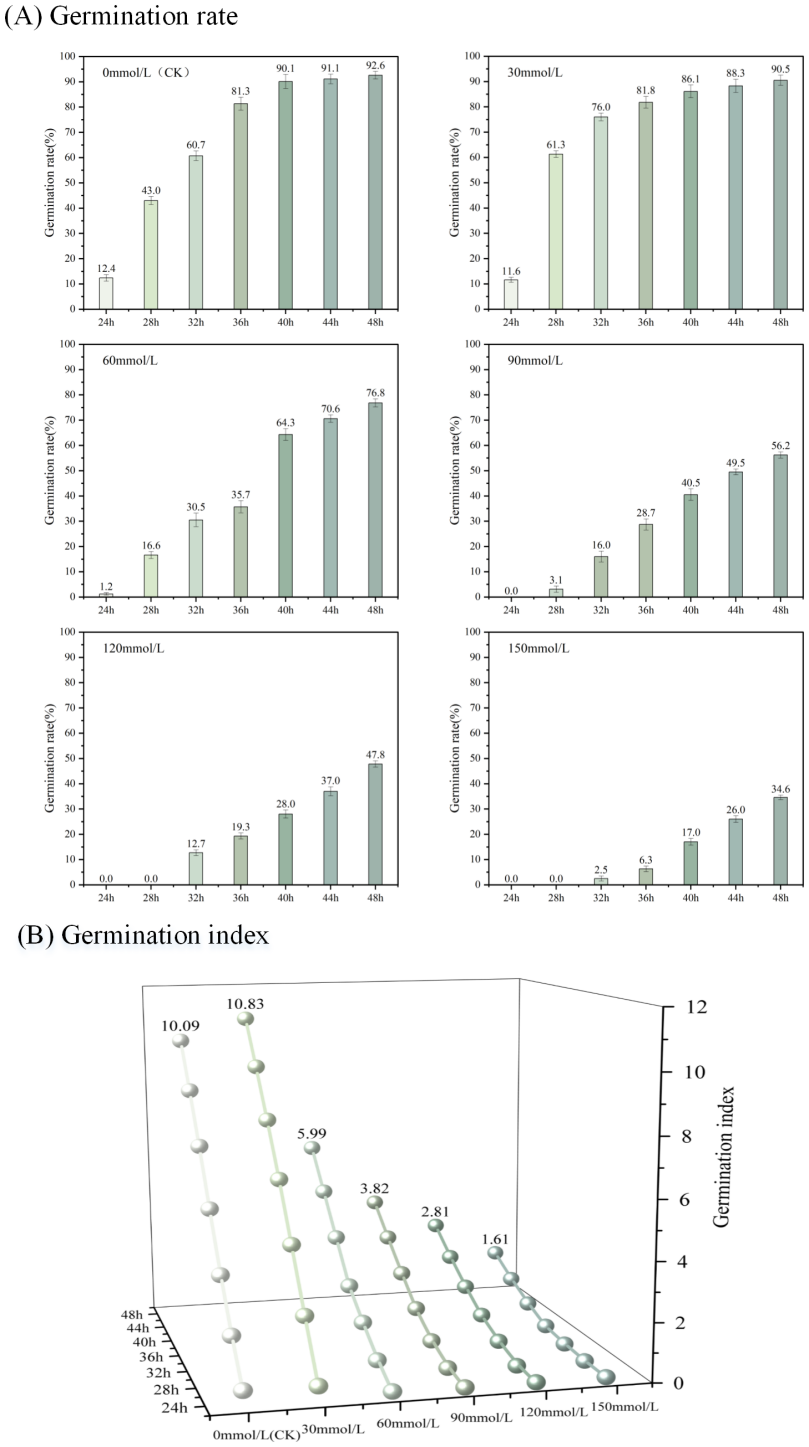
Trends of germination rate and germination index in deionized water and different concentrations of NaCl solution. **(A)** Shows the germination rate and **(B)** shows the germination index.

**Figure 10A** demonstrates the germination rate of cucumber seeds sprouting in deionized water (CK) as well as in different concentrations of NaCl solution over time. The germination rate gradually decreased at each time point with the increase in the concentration (60, 90, 120, and 150 mmol/L) of NaCl solution. However, the germination rate was significantly higher than that of other concentrations and higher than that of the control group at 28–36 h at 30 mmol/L. **Figure 10B** shows the results of the cucumber seed germination index in deionized water (CK) and different concentrations of NaCl solution over time. At each time point, the germination index in the 30 mmol/L NaCl solution was higher than those in the other concentrations and CK. As the concentration of NaCl solution increased, the germination index gradually decreased. In general, NaCl solution has an inhibitory effect on the germination of cucumber seeds. The higher the concentration, the more pronounced the inhibitory effect, but at a certain germination time period, a certain concentration of NaCl solution could promote the germination of cucumber seeds.

**Figure 11:**
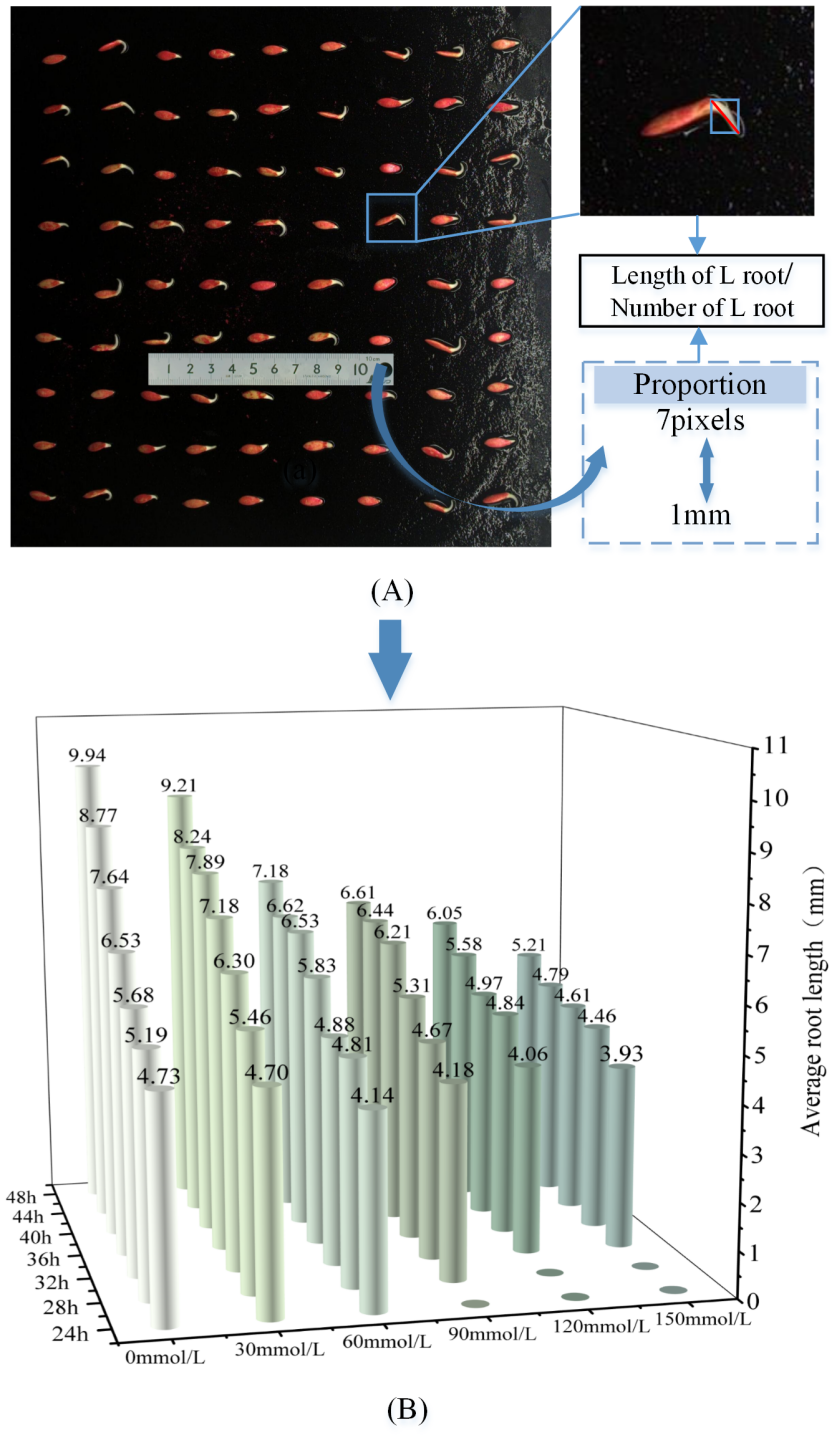
**(A)** Schematic diagram of root length measurement **(B)** Changes in root length of cucumber sprouting in different concentration solutions.

Extract all L root targets detected by the YOLOv8-ECS model targets as shown in **Figure 11A**, approximate the root length characteristics during cucumber seed germination with the diagonal length of the detection frame, converted the pixel points into lengths proportionally, and added the extracted root length data. We then divided them by the number of sprouted seeds to obtain the average root lengths of seed germinated under different concentrations of NaCl solution and determine the characteristics of the changes over time. A 3D histogram is shown in **Figure 11B**. The characteristics of root length and germination rate of cucumber seed germination under different concentrations of NaCl solution were consistent with the role of promoting root elongation under 30 mmol/L NaCl solution. High concentrations of NaCl solution could inhibit root elongation; as the concentration increased, the inhibition of root elongation became more obvious.

## 4 Discussion

To explore the germination vigor of cucumber seeds under salt stress environment and solve the drawbacks of traditional manual monitoring of seed germination vigor index, we carried out a germination test on cucumber seeds under salt stress environment based on the seed germination phenotype collection system and obtained the germination images. Based on the YOLOv8-n model, the YOLOv8-ECS model applicable to the detection of cucumber seed germination state was proposed, and the germination state detection was carried out on the captured images of cucumber seed germination, and the germination vitality index of cucumber seed germination was analyzed to obtain the cucumber seed germination vitality index under the salt stress environment, which provides a convenient and fast new method for selecting and breeding salt-tolerant cucumber varieties. The main tasks of the model were as follows:

1. Based on the seed germination phenotype acquisition system, the system mainly consisted of three parts: seed germination culture bin, image acquisition system, and human–computer interaction module, which realized the function of phenotypic dynamic acquisition of the complete cycle of seed germination in three stations. The cucumber seed germination test was carried out in the system under the stress environment of five concentrations of NaCl solution. The images were collected once every 30 min during the germination process. A total of 1728 germination images were obtained, and a continuous process dataset of cucumber seed germination was constructed.
2. By comparing the detection accuracy of different models, YOLOv8-n was selected as the base model, and the original loss function was replaced by ECIoU_Loss loss function. CA mechanism was added to the head, and small-target detection head was added for tiny targets. The YOLOv8-ECS model was obtained. The precision P, recall R, and mAP of the improved model increased from the original 91.6%, 85.4%, and 91.8% to 96.9%, 97.3%, and 98.9%, respectively. Compared with the original model, the model volume increased slightly, which effectively improved the model’s ability to recognize the phenotypic features of cucumber seed germination.
3. The germination rate, germination index, and root length as the germination vigor index were detected. We analyzed the germination images through the YOLOv8-ECS model. The cucumber germination rate, germination index, and root length were obtained under different salt solution environments. A graph of the change in each parameter over time was drawn to determine the characteristics of cucumber seed germination in a salt solution environment. The data showed that a low concentration of salt solution could promote the germination of cucumber seeds, and the seed germination vigor gradually decreased with the increase in salt concentration. Thus, a high concentration of salt solution had an inhibitory effect on the germination of cucumber seeds. The higher the concentration, the stronger the inhibitory effect.

Through the comparative experiments we have done above, it can be seen that the detection accuracy of the YOLOv8-ECS model developed by us is higher than that of other models, and its mAP reaches 98.9%, which greatly improves the detection performance and reduces missed detections and repeated detections. At the same time, compared with the methods used by Fu et al. (2022) and Jiang et al. (2023) cited above, our method can roughly calculate the root length characteristics of the seed germination process, and use more seed germination vigor characteristics to evaluate the germination vigor of seeds, making our seed germination vigor assessment method more realistic and reliable.

However, the model we developed has some limitations. The model will have a small amount of misjudgment for the detection of long and short roots, probably because the model is not sensitive enough to the length information and cannot well understand the sprouting judgment criteria in terms of length; on the other hand, we focused on the detection accuracy of the model when choosing the model evaluation index, which resulted in the increased size of the improved model, which is not conducive to the deployment of the practical application in the later stage.

In the future, we will further improve the performance of the seed germination phenotype collection system, embed the model into the germination system, realize the real-time monitoring and recording of the germination vigor of seeds, and explore the other phenotypic characteristics in the germination of cucumber seeds. Such work will allow us to provide an accurate, efficient, and quick method for the selection and breeding of good varieties.

## Data Availability

The raw data supporting the conclusions of this article will be made available by the authors, without undue reservation.
